# Longitudinal ecological disruption and metabolite associations of the gut microbiome during acute myeloid leukemia therapy

**DOI:** 10.1128/spectrum.02578-26

**Published:** 2026-09-08

**Authors:** Jian Zhou, Rong Li, Mengyue Zhang, Yong Wu, Rong Hu

**Affiliations:** 1 The School of Nursing, Fujian Medical University 74551 , Fuzhou, Fujian, China; 2 Fujian Provincial Key Laboratory on Hematology, Fujian Institute of Hematology, Fujian Medical University Union Hospital 117890 , Fuzhou, Fujian, China; Cleveland Clinic Lerner Research Institute, Cleveland, Ohio, USA

**Keywords:** acute myeloid leukemia, gut microbiome, ecological disruption, antibiotic exposure, short-chain fatty acids, metabolomics

## Abstract

Acute myeloid leukemia therapy is accompanied by antibiotic exposure and marked gut microbiome disruption. We analyzed 508 longitudinal stool 16S rRNA gene profiles from 67 patients in PRJNA451154. We summarized the depletion of putative short-chain fatty acid (SCFA)-associated genera, pathobiont expansion, and domination with an ecological composite score (ECS). Disruption peaked descriptively during days 15–28 (D15–D28). In a patient-clustered generalized estimating equation model jointly including treatment window and antibiotic exposure during the preceding 0–14 days, the D15–D28 ECS contrast was attenuated (coefficient, 0.251; 95% confidence interval [CI], −0.372 to 0.874), whereas recent antibiotic exposure remained associated with higher ECS (0.722 per 7 antibiotic-days; 95% CI, 0.529 to 0.915). The D1–D7 to D15–D28 increase persisted with patient-level fivefold cross-fitted score construction (paired difference, 1.279; bootstrap 95% CI, 0.861 to 1.758). Treatment-window-adjusted models retained associations of serum butyrate/isobutyrate with the putative SCFA-associated genus signal (0.476; 95% CI, 0.309 to 0.643) and fixed ECS (−0.372; 95% CI, −0.516 to −0.227). ECS did not add stable information beyond Shannon loss and maximum pathobiont abundance for clinical events during the subsequent 7 or 14 days. Frozen ecological formulas produced similar rankings across two external data sets but did not provide clinical validation. Thus, D15–D28 was a descriptive window strongly coupled to recent antibiotic exposure, and ECS functioned as an integrative ecological summary rather than a validated predictor.

**IMPORTANCE:**

Acute myeloid leukemia therapy creates a clinically important perturbation of gut bacterial communities, yet microbiome injury is often summarized by diversity alone or by isolated taxa. We integrated the depletion of genera associated with short-chain fatty acid production and expansion of potential pathogens into a longitudinal ecological score. The apparent disruption peak during days 15–28 was largely attenuated after accounting for recent antibiotic exposure, whereas patient-level resampling supported the stability of the ecological contrast. Associations with serum metabolites persisted after adjustment for treatment period, but amplicon sequencing cannot establish microbial function or metabolite production. The composite score did not add stable information beyond diversity loss and maximum potential-pathogen abundance for near-term clinical events. These findings define a reproducible framework and a high-yield sampling window for prospective studies while avoiding causal or predictive claims.

## INTRODUCTION

Patients receiving intensive therapy for acute myeloid leukemia (AML) enter a period of high infectious risk, frequent antibiotic exposure, and mucosal injury (1–3). Longitudinal AML data sets also show that this treatment period is accompanied by disruption of host-microbial homeostasis (4, 5). The gut microbiome is a plausible contributor to this risk because it helps maintain colonization resistance and restrain pathobiont expansion (6, 7). It also shapes microbial metabolite availability and mucosal immune tone (8, 9). In this setting, microbiome injury is not only a question of which organisms are present. It is also a question of when the community enters a vulnerable ecological state.

Prior AML and leukemia studies have linked induction chemotherapy with reduced microbial diversity and infectious complications (2, 4, 10–12). In related hematopoietic-cell transplantation settings, low diversity and intestinal domination by potential pathogens have been associated with bacteremia, graft-versus-host disease, and mortality (13–15). Antibiotic-focused studies further show that microbiome disruption can be individualized, persistent, and associated with incomplete recovery after exposure (16–18). These clinical observations fit a broader biological framework in which short-chain fatty acids (SCFAs), indole derivatives, and bile acid pathways connect microbial community structure to barrier and immune biology (8, 19–21). Together, these findings argue that treatment-period microbiome disruption has both compositional and metabolic dimensions. Yet many analyses still rely on cross-sectional sampling, single-endpoint framing, or outcome-first models that do not first define the ecological state being measured.

An integrated analytical framework should first characterize longitudinal gut ecological disruption during therapy and then test whether treatment timing and recent antibiotic exposure contribute independent information. It should also assess whether score construction is stable when scaling parameters are estimated without the patient being evaluated. Further analyses should determine whether metabolite associations persist after treatment-window adjustment and whether ECS adds clinical information beyond established component metrics. Each data set should be judged according to the metadata it provides. A discovery cohort with linked microbiome, antibiotic, clinical-event, and metabolomic data can support integrated modeling. Public data sets with narrower metadata can support application of fixed rules, distributional comparisons, or assessment of metabolite directionality, but not clinical validation.

Here, we analyzed longitudinal stool 16S amplicon profiles from an AML discovery cohort and defined a genus-level ecological composite score based on the depletion of putative SCFA-associated genera and expansion of prespecified pathobionts. We jointly modeled treatment window and recent antibiotic exposure, evaluated patient-level cross-fitted score construction and bootstrap stability, and adjusted metabolite-ecology associations for treatment window. We also assessed informative sampling and directly compared ECS with Shannon diversity and maximum pathobiont abundance for subsequent clinical events. External stool microbiome data sets were used only to evaluate a frozen ECS formula and a fixed compositional log-ratio. A fecal metabolite data set provided exploratory directional support. This design treats ecological disruption as an operational, potentially reversible state and keeps causal, functional, predictive, and validation claims within the limits of the available data.

## RESULTS

### The discovery and external data sets were assigned distinct analytical roles

We organized the analysis around the claims each data set could support (Fig. 1). PRJNA451154 served as the discovery cohort because it linked longitudinal stool microbiome profiles with antibiotic exposure, clinical-event metadata, and serum metabolomic readouts. The main analysis included 508 adequate stool samples from 67 patients. Contributions by treatment window were PreChemo, 29 samples from 25 patients; D1–D7, 140 from 62; D8–D14, 122 from 61; D15–D28, 193 from 56; and PostD28, 24 from 18. Patients contributed a median of 8 stool samples (range, 1–14); the full patient-level distribution is reported in Table S1. Serum-stool pairing yielded 227 nearest pairs within 3 days and 243 pairs within 7 days from 35 patients.

**Fig 1:**
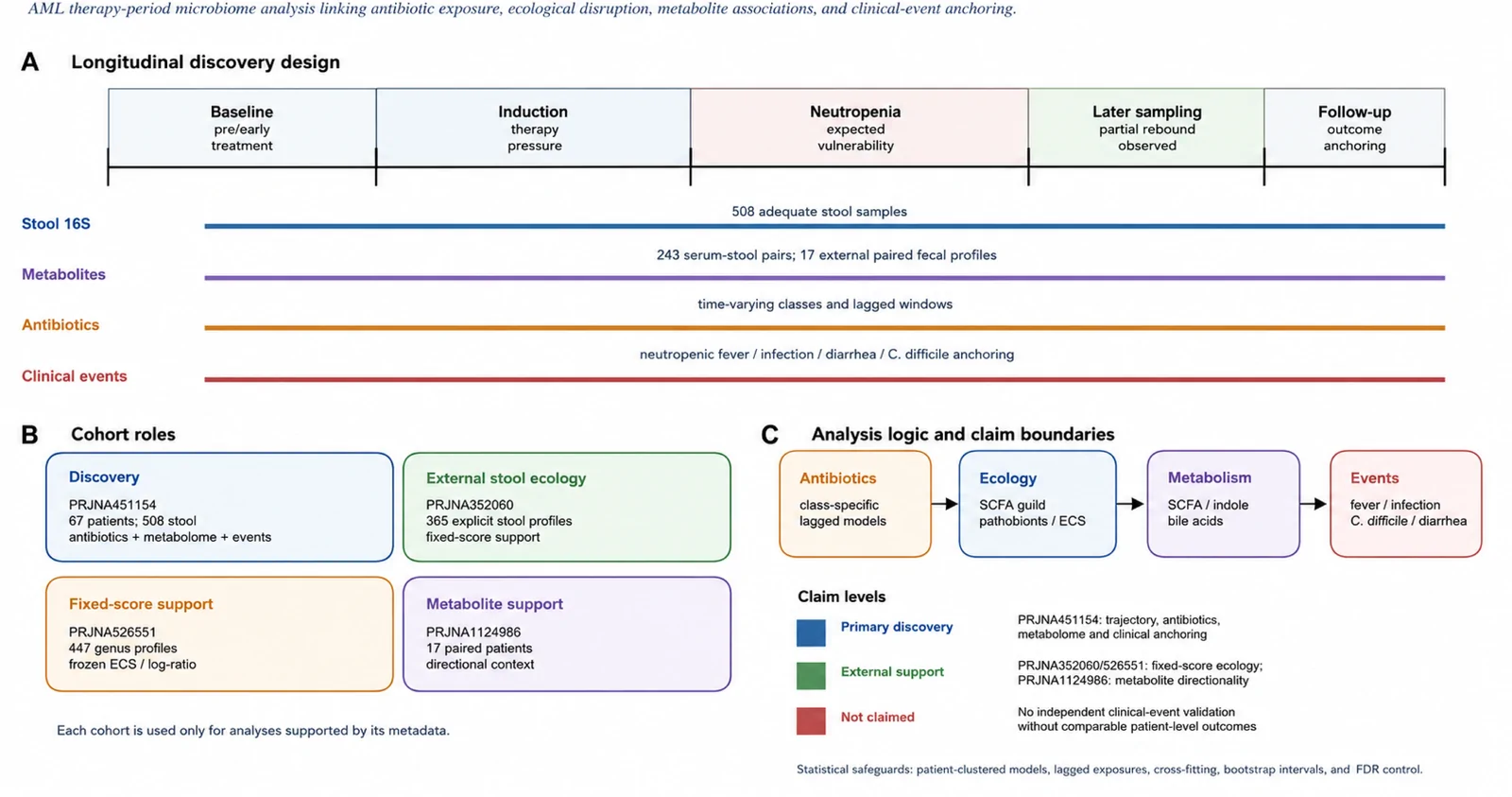
Study design and analytical framework for longitudinal gut microbiome analysis during AML therapy. (**A**) Longitudinal discovery design showing the AML therapy-period timeline and the main data streams used for stool 16S microbiome profiling, serum metabolomics, antibiotic exposure modeling, and clinical-event anchoring. The discovery analysis included 508 adequate stool samples, 243 serum-stool pairs within 7 days, and time-varying antibiotic exposure classes. (**B**) Cohort roles were assigned according to the available metadata. PRJNA451154 served as the discovery cohort. PRJNA352060 and PRJNA526551 were used for external ecological support, and PRJNA1124986 was used for patient-linked fecal metabolite support. (**C**) Analysis logic linking antibiotic exposure, ecological indices, metabolite readouts, and clinical-event anchoring. The claim levels distinguish primary discovery, external support, and endpoints not claimed. External data sets were not treated as independent validation cohorts for neutropenic fever, infection, C. difficile infection, or antibiotic effects.

External data sets were assigned narrower roles. PRJNA352060 and PRJNA526551 were used to evaluate whether the frozen ECS formula and a prespecified compositional log-ratio could be computed without cohort-specific recalibration. PRJNA1124986 was used only for patient-linked fecal metabolite directionality. These data sets were not pooled with PRJNA451154 and were not treated as biological or clinical validation cohorts because they did not provide the same treatment-day, antibiotic, and clinical-event structure.

### Ecological disruption metrics showed a descriptive peak during D15–D28

Ecological disruption metrics changed across the AML treatment timeline (Fig. 2). Median abundance of putative SCFA-associated genera was highest during D1–D7 (0.081) and fell by D15–D28 (0.003). Over the same interval, median pathobiont burden increased from 0.024 to 0.063, median maximum pathobiont abundance increased from 0.021 to 0.050, and median ECS rose from −0.410 to 0.921. These unadjusted window summaries describe temporal concentration of disruption but do not distinguish treatment time from accumulating antibiotic exposure.

**Fig 2:**
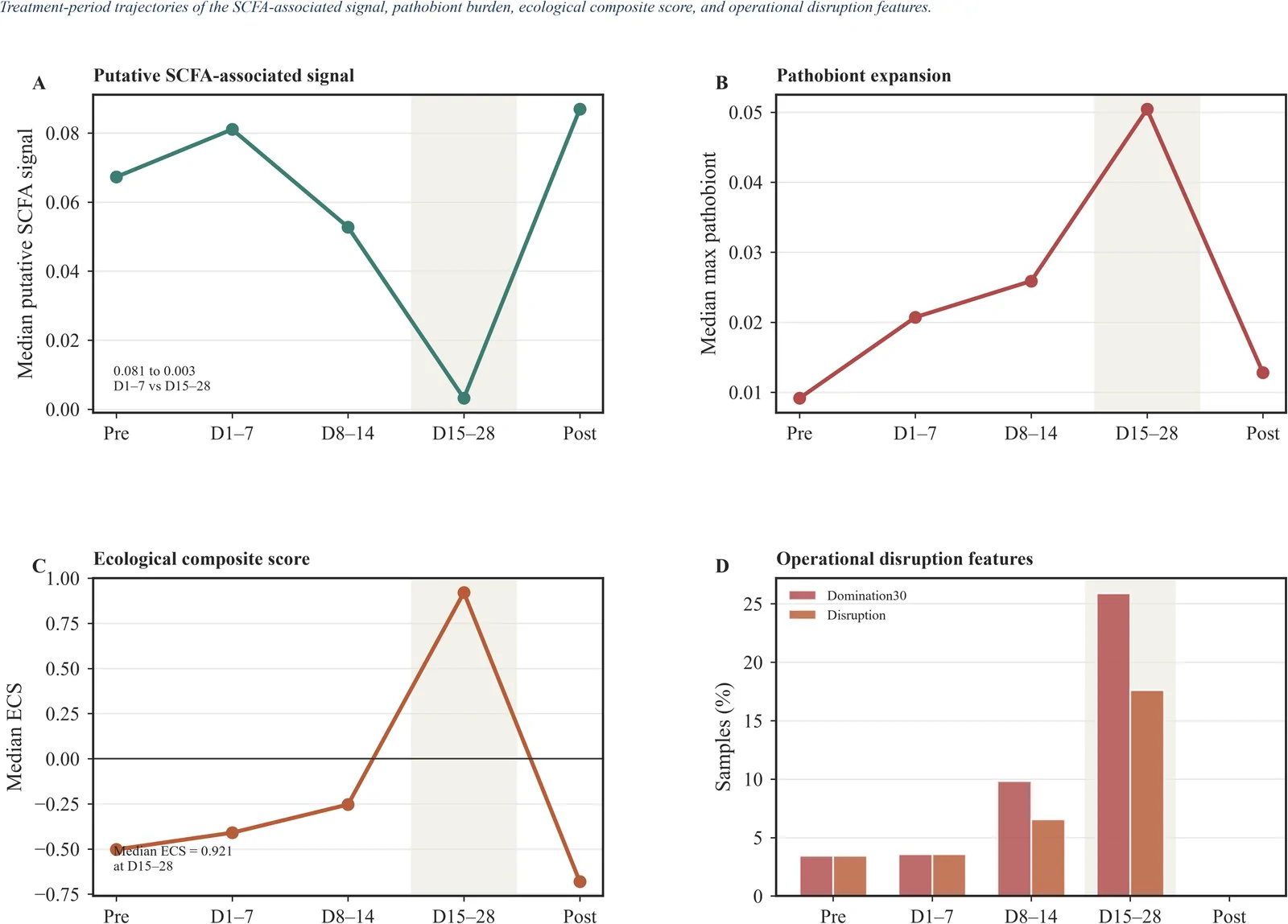
Longitudinal ecological disruption metrics show a descriptive peak during D15–D28 of AML therapy. (**A**) Median abundance of putative SCFA-associated genera across therapy windows. (**B**) Median maximum pathobiont abundance. (**C**) Median ecological composite score (ECS). (**D**) Domination30 and the operational ecological-disruption rate. Contributions were PreChemo, 29 samples/25 patients; D1–D7, 140/62; D8–D14, 122/61; D15–D28, 193/56; and PostD28, 24/18. The shaded interval marks the descriptive D15–D28 peak. These unadjusted panels do not distinguish treatment timing from recent antibiotic exposure, and any apparent PostD28 recovery applies only to the available samples.

Binary disruption features followed the same descriptive pattern. Domination30 increased from 3.6% during D1–D7 to 25.9% during D15–D28, and the prespecified disruption-state rate increased from 3.6% to 17.6%. Among the 24 PostD28 samples available from 18 patients, median ECS was lower (−0.680), no Domination30 events were observed, and median putative SCFA-associated genus abundance was higher (0.087). This is compatible with partial recovery among available samples, but the small and selected PostD28 subset does not establish cohort-wide recovery.

The within-patient D15–D28 minus D1–D7 contrast was evaluated among 52 patients contributing samples to both windows (Fig. S1). The fixed ECS paired difference was 1.973 (2,000-resample patient-bootstrap 95% CI, 1.318 to 2.667). When robust centers and scales were estimated in five patient-level training folds and applied only to held-out patients, the cross-fitted ECS remained strongly correlated with the fixed ECS (Spearman rho, 0.957) and retained a paired difference of 1.279 (95% CI, 0.861 to 1.758). A prespecified pathobiont-minus-SCFA compositional log-ratio also tracked fixed ECS in the discovery cohort (rho, 0.843). These analyses support score stability but do not remove time-varying exposure confounding.

### Diversity and community structure shifted during the disruption window

The D15–D28 pattern was accompanied by broader community disruption rather than only a change in the fixed putative SCFA-associated and pathobiont sets (Fig. 3). Shannon diversity declined during D15–D28 compared with D1–D7 (median, 2.05 vs 2.50) and PreChemo (2.05 vs 2.70). Effective genera fell from a median of 12.17 during D1–D7 to 7.77 during D15–D28, while richness at the 0.1% threshold decreased from 34 to 20 genera. Patient-level bootstrap estimates for the D15–D28 minus D1–D7 Shannon contrast were −0.535 (95% CI, −0.767 to −0.315).

**Fig 3:**
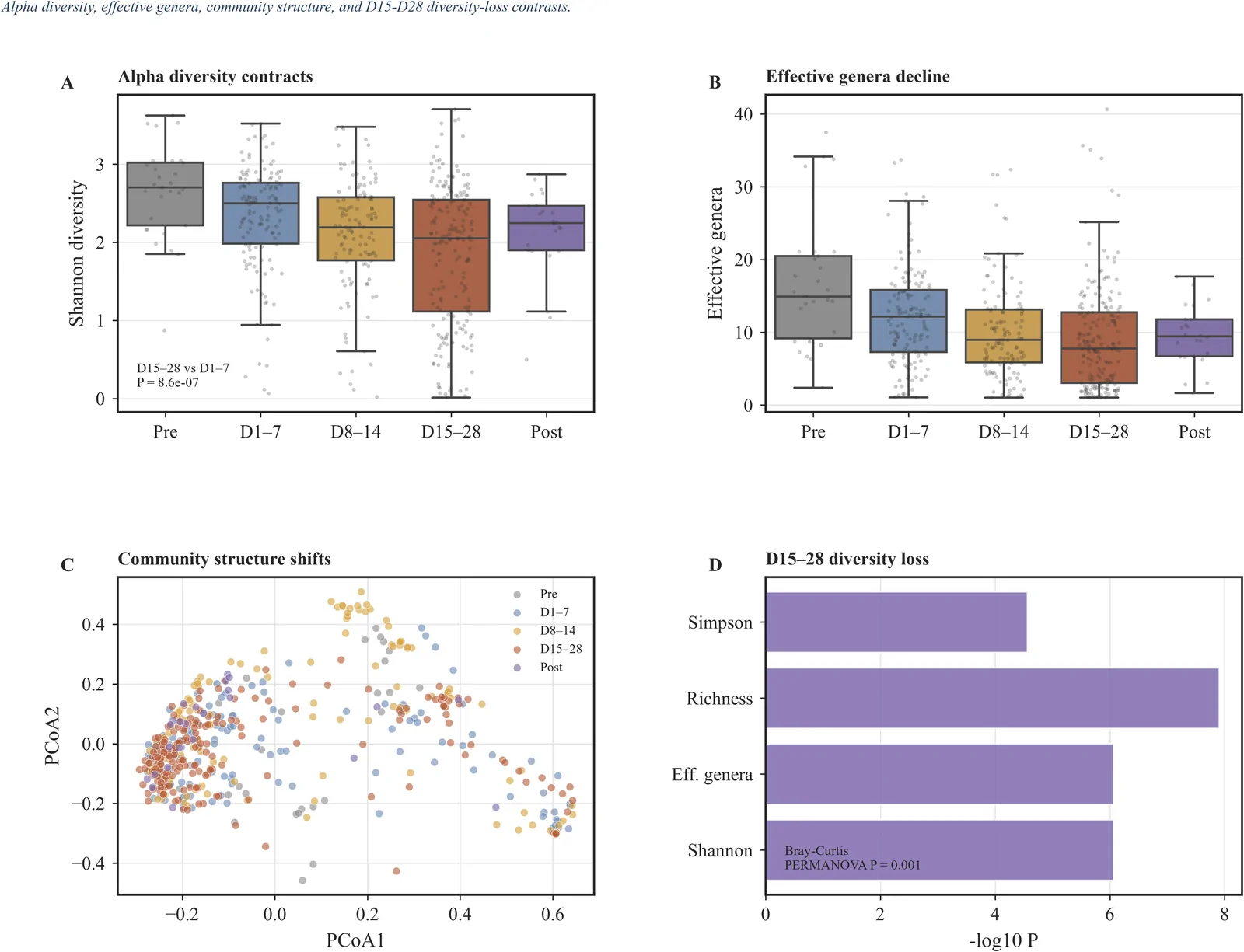
Ecological disruption is accompanied by community-wide diversity and structure shifts. (**A**) Shannon diversity across treatment windows. (**B**) Effective genera across treatment windows. (**C**) Bray-Curtis PCoA showing community structure by therapy period. (**D**) Summary of D15–D28 diversity contrasts and the Bray-Curtis PERMANOVA result (*P* = 0.001; 999 treatment-period label permutations restricted within patient). The figure places the putative SCFA-associated/pathobiont/ECS signal in a broader community context. Because the analysis is genus-level 16S, these results should not be interpreted as strain-level replacement, function, or direct pathogen identification.

Community composition also differed by treatment period. Bray-Curtis ordination showed period-associated displacement. Global PERMANOVA indicated period-level differences in beta diversity under both unrestricted and within-patient restricted label permutations (pseudo-*F* = 5.089; *P* = 0.001 for each scheme; 999 permutations per scheme). Together, the alpha- and beta-diversity results placed the D15–D28 signal in a community-wide rather than single-marker context.

### The D15–D28 ecological contrast attenuated after adjustment for recent antibiotic exposure

Recent antibiotic exposure increased across the main treatment windows and was strongly coupled to treatment time (Fig. 4). Median exposure during the preceding 0−14 days was 7 antibiotic-days at D1–D7, 16 at D8–D14, and 23 at D15–D28; one-way treatment-window eta-squared was 47.9% for sample-level recent exposure. In period-only patient-clustered GEE, D15–D28 was associated with higher fixed ECS relative to D1–D7 (coefficient, 1.812; 95% CI, 1.260 to 2.364). After joint inclusion of recent exposure, the D15–D28 coefficient attenuated to 0.251 (95% CI, −0.372 to 0.874), whereas recent exposure remained associated with higher ECS (0.722 per 7 antibiotic-days; 95% CI, 0.529 to 0.915).

**Fig 4:**
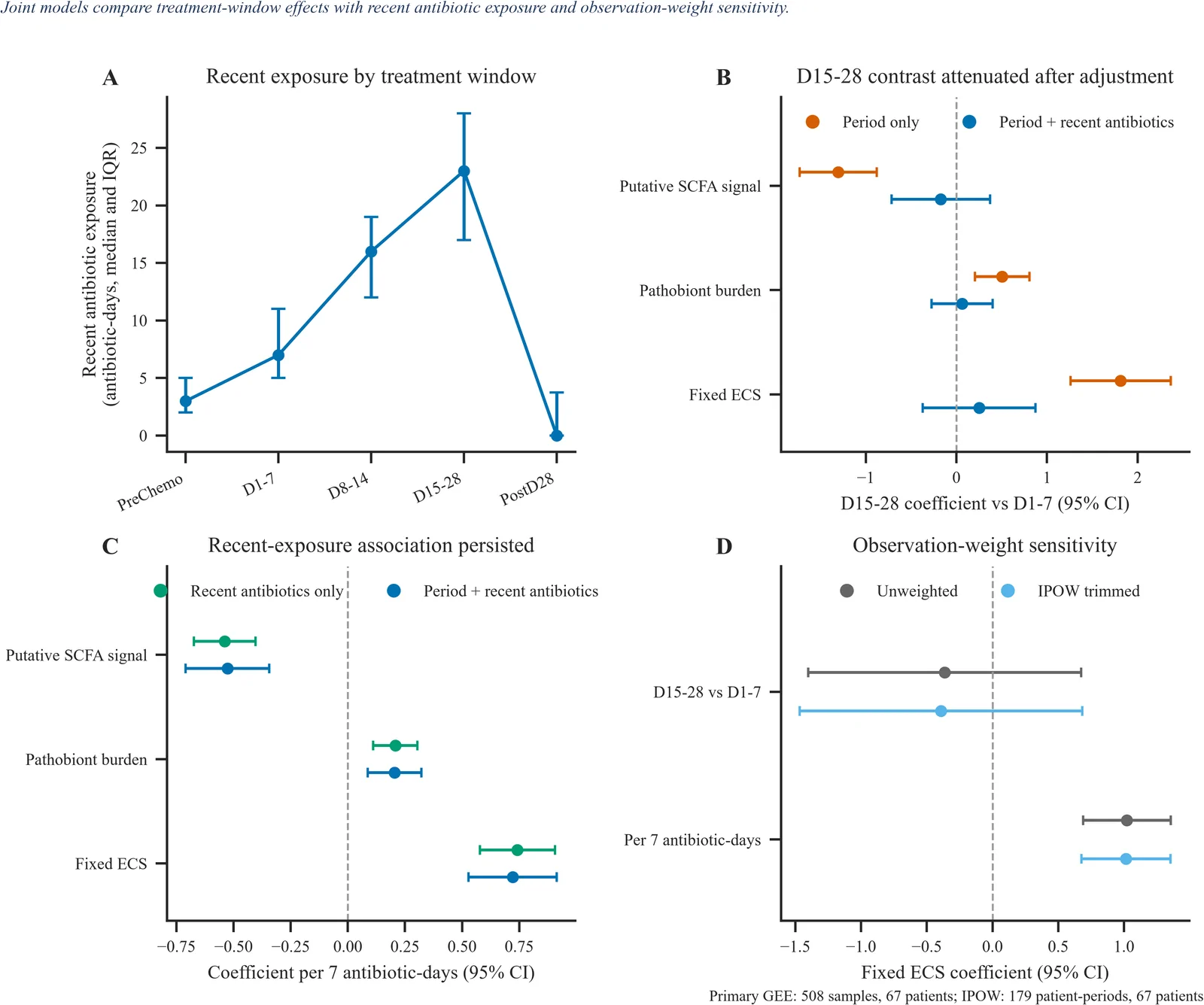
Joint models distinguish treatment-window and recent-antibiotic associations. (**A**) Recent antibiotic exposure during the preceding 0−14 days by treatment window, shown as the median and interquartile range of antibiotic-days. (**B**) D15–D28 vs D1–D7 coefficients from treatment-window-only and joint treatment-window plus recent-antibiotic GEE models for the putative SCFA signal, pathobiont burden, and fixed ECS. (**C**) Coefficients per 7 recent antibiotic-days from exposure-only and joint models for the same three ecological outcomes. (**D**) Unweighted and trimmed inverse-probability-of-observation-weighted estimates for fixed ECS. Points show coefficients and lines show robust 95% confidence intervals. Primary models include 508 samples from 67 independent patient clusters; the observation-weighted analysis includes 179 observed patient-periods from 67 patients. Attenuation of the D15–D28 contrast after adjustment, together with persistence of the recent-exposure association, does not establish antibiotic causality.

The same joint model showed attenuation of the D15–D28 contrasts for log-transformed putative SCFA-associated abundance (coefficient, −0.172; 95% CI, −0.715 to 0.371) and log-pathobiont burden (0.063; 95% CI, −0.274 to 0.401). In contrast, recent exposure retained associations with lower log-SCFA (−0.527 per 7 antibiotic-days; 95% CI, −0.710 to −0.344) and higher log-pathobiont burden (0.205; 95% CI, 0.087 to 0.322). All models included 508 observations and 67 independent patient clusters and used robust sandwich covariance. Results were directionally consistent with an independence working correlation and in 2,000 patient-level bootstrap refits. Thus, D15–D28 is best interpreted as a descriptive disruption window strongly coupled to recent antibiotic exposure, not as an exposure-independent therapy-period state.

We next assessed whether observation frequency could materially alter this conclusion (Fig. S2). Among samples eligible for repeat observation, the probability of another stool sample within 7 days was 95.7% at D1–D7, 90.2% at D8–D14, and 88.3% at D15–D28. Stabilized inverse-probability-of-observation weights showed acceptable positivity (denominator probability range, 0.400−0.992; effective sample size, 177.8 of 179 observed patient-periods). In weighted joint models, the D15–D28 fixed-ECS contrast remained attenuated (coefficient, −0.392; 95% CI, −1.468 to 0.683), whereas recent antibiotic exposure remained associated with higher ECS (1.014 per seven antibiotic-days; 95% CI, 0.676 to 1.353). This proxy analysis cannot recover unrecorded eligibility after discharge, death, transfer, or protocol completion.

### Serum metabolite associations persisted after treatment-window adjustment

Serum metabolite associations were re-estimated with adjustment for treatment window to reduce confounding by shared temporal trajectories (Fig. 5). The primary analysis included 243 nearest serum-stool pairs within 7 days from 35 patients and used rank-normalized variables in patient-clustered GEE. Butyrate/isobutyrate remained positively associated with the putative SCFA-associated genus signal (coefficient, 0.476; 95% CI, 0.309 to 0.643; FDR, 4.56e−06) and negatively associated with fixed ECS (−0.372; 95% CI, −0.516 to −0.227; FDR, 5.17e−05). Corresponding 3-day-window sensitivity estimates were 0.472 (95% CI, 0.306 to 0.639) and −0.374 (95% CI, −0.528 to −0.220). See Fig. S5.

**Fig 5:**
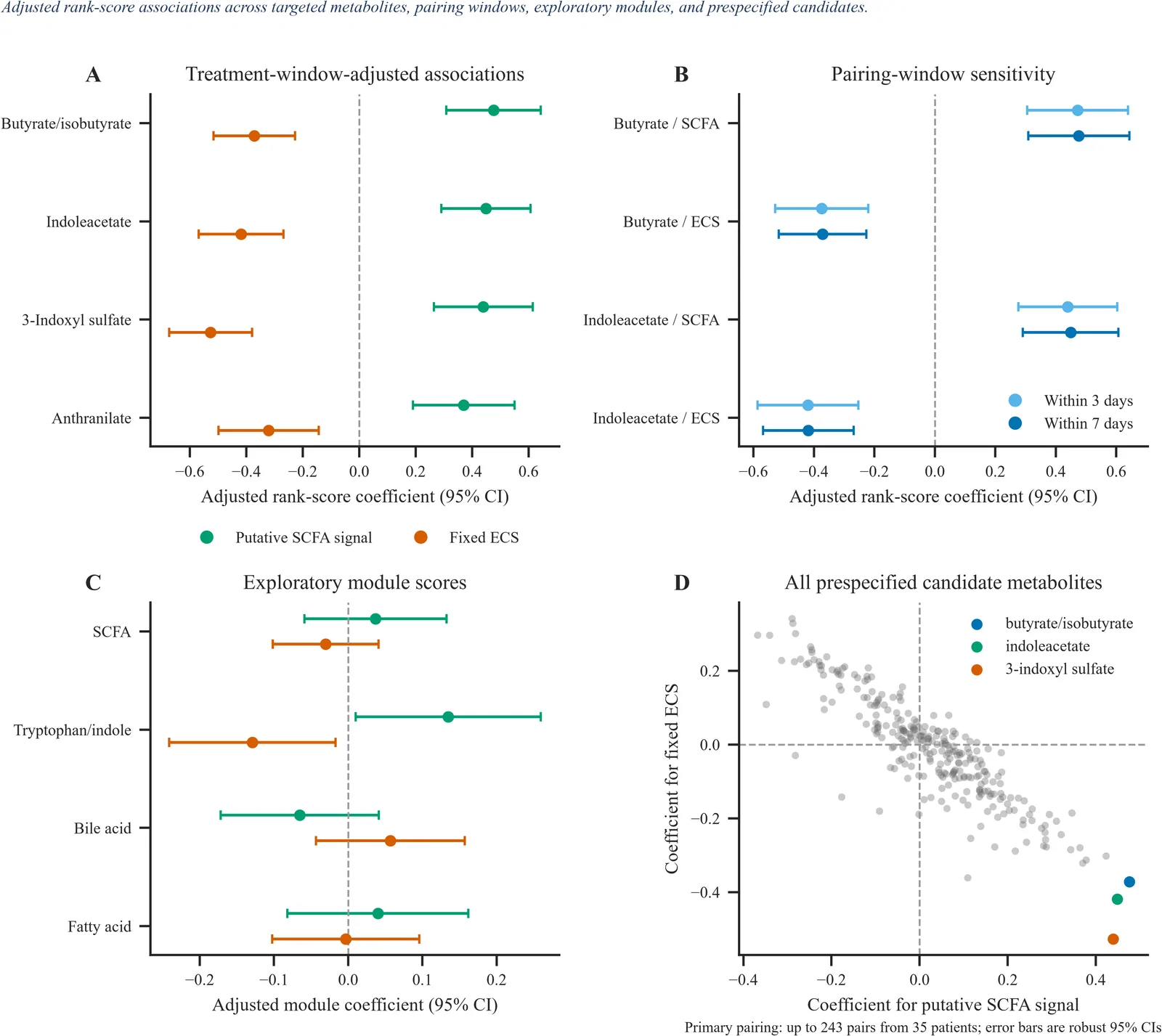
Serum metabolite modules align with stool ecological state. (**A**) Treatment-window-adjusted associations of butyrate/isobutyrate, indoleacetate, 3-indoxyl sulfate, and anthranilate with the putative SCFA signal and fixed ECS in nearest serum-stool pairs within 7 days. (**B**) Sensitivity of the butyrate/isobutyrate and indoleacetate estimates to pairing within 3 vs 7 days. (**C**) Exploratory associations of prespecified serum metabolite-module scores with the two ecological indices. (**D**) Coefficients for all prespecified candidate metabolites, comparing association with the putative SCFA signal on the *x* axis and fixed ECS on the *y* axis; highlighted points denote the targeted metabolites shown in panel A. Points show rank-score coefficients, and lines show patient-clustered robust 95% confidence intervals from treatment-window-adjusted models. The primary pairing provided up to 243 pairs from 35 patients. Serum metabolites are systemic correlates and do not establish microbial production, metabolic flux, or causality.

Window-adjusted associations were also retained for prespecified indole-related metabolites. Indoleacetate was positively associated with the putative SCFA-associated genus signal (coefficient, 0.449; 95% CI, 0.291 to 0.607) and negatively associated with fixed ECS (−0.419; 95% CI, −0.569 to −0.268). Similarly, 3-indoxyl sulfate was positively associated with the putative SCFA-associated signal (0.440; 95% CI, 0.264 to 0.615) and negatively associated with fixed ECS (−0.527; 95% CI, −0.674 to −0.379). These results indicate covariation beyond coarse treatment-window adjustment but do not establish microbial production, metabolic flux, or causality.

### Clinical anchoring analyses showed no incremental information from ECS

Internally derived percentile rules summarized sample-level ecology into descriptive patient trajectories (Fig. S3): 33 patients were assigned to Stratum A, 6 to Stratum B, and 17 to Stratum C; 7 had no threshold signal, and 4 had sparse data. In the descriptive display, the latter 11 patients are shown together as no threshold signal. As expected from their definitions, these strata differed in maximum ECS, minimum putative SCFA-associated abundance, maximum pathobiont abundance, and the timing of extreme samples. Because the rules and thresholds were derived from the same cohort, these differences are descriptive and cannot be interpreted as severity, discrimination, calibration, or validation.

Peak timing in the descriptive strata concentrated in the same unadjusted treatment window identified in the sample-level analysis. In Stratum A, 22 of 33 patients had peak ECS during D15–D28, as did all 6 patients in Stratum B. This temporal concentration should be interpreted alongside the joint exposure models, in which adjustment for recent antibiotic exposure substantially attenuated the D15–D28 contrast.

For a non-self-referential clinical anchor, we compared cross-fitted ECS, fixed ECS, Shannon loss, and maximum pathobiont abundance for first recorded C. difficile infection, diarrhea, or neutropenic fever during the subsequent 7 or 14 days (Fig. 6). Models excluded samples collected on or after each patient’s first event and adjusted for treatment window. Cross-fitted ECS showed no stable association with subsequent events; for C. difficile infection, odds ratios per rank-normalized SD were 1.128 (patient-bootstrap 95% CI, 0.651 to 1.914) at 7 days and 1.231 (95% CI, 0.663 to 2.207) at 14 days. Shannon loss showed stronger nominal associations before multiplicity correction for subsequent C. difficile infection (7-day OR, 1.659; 95% CI, 1.101 to 2.620; 14-day OR, 1.880; 95% CI, 1.195 to 3.137).

**Fig 6:**
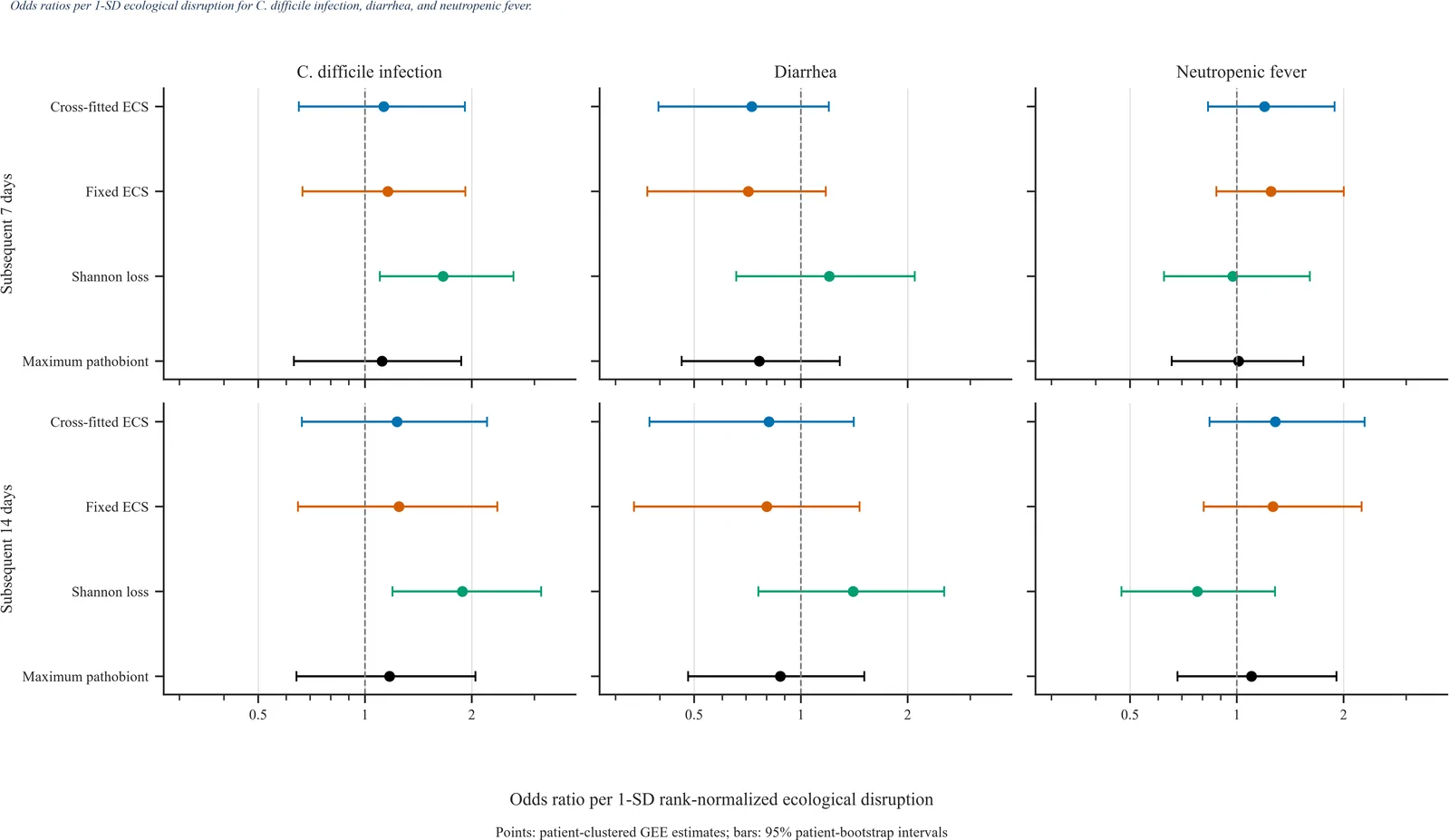
Direct comparison of ecological metrics for subsequent clinical events Period-adjusted odds ratios and patient-bootstrap 95% confidence intervals compare cross-fitted ECS, fixed ECS, Shannon loss, and maximum pathobiont abundance for the first recorded C. difficile infection, diarrhea, or neutropenic fever during the subsequent 7 or 14 days. Samples on or after a patient’s first event were excluded for that outcome, and all metrics were rank-normalized and oriented, so higher values indicate disruption. No comparison met an FDR threshold of 0.05, and ECS did not show stable incremental information beyond Shannon loss and maximum pathobiont abundance. These analyses provide clinical anchoring, not validated predictive performance.

None of the 24 primary metric-event-horizon comparisons met an FDR threshold of 0.05. In exploratory incremental models already containing Shannon loss and maximum pathobiont abundance, adding cross-fitted or fixed ECS did not provide stable incremental information across outcomes and horizons. The analysis, therefore, does not support superiority of ECS over its constituent or established ecological metrics and should be interpreted as clinical anchoring rather than predictive-model validation.

### Fixed ecological formulas could be applied across external genus tables without recalibration

We applied the same prespecified genus sets, ECS pseudocount, compositional zero replacement, and thresholds to the 508-sample discovery table, 447 PRJNA526551 profiles, and 365 explicit PRJNA352060 stool profiles without external recalibration (Fig. 7; Fig. S4). Fixed ECS correlated with the fixed mean-log pathobiont-minus-SCFA contrast in the discovery cohort (Spearman rho, 0.843), PRJNA526551 (rho, 0.870), and PRJNA352060 (rho, 0.857). Sample ordering was, therefore, similar under two prespecified representations of the same taxa—a log ratio of summed abundances and a genus-wise mean-log contrast—but this agreement is not independent biological validation.

**Fig 7:**
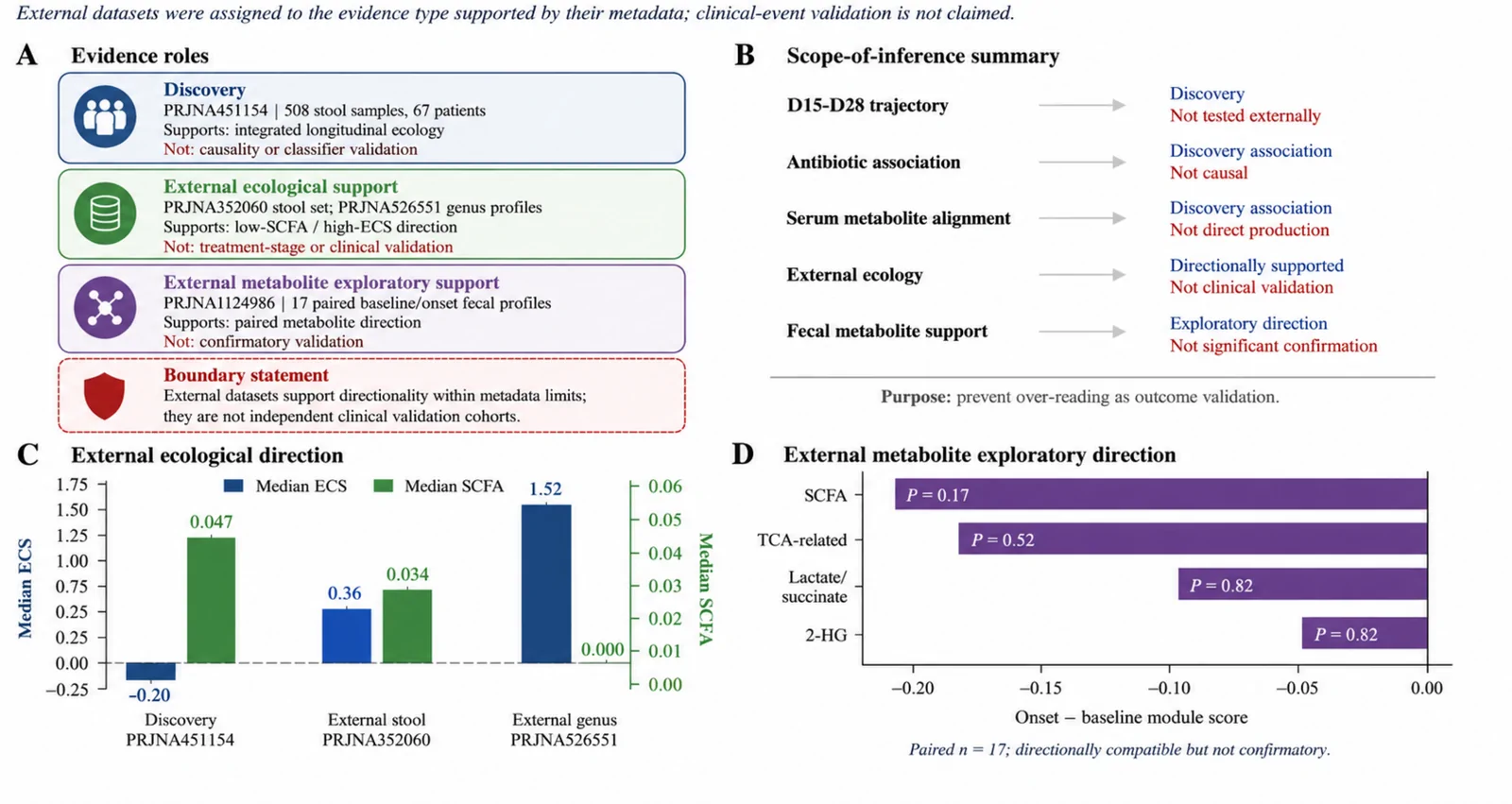
Boundaries of inference for external ecological and metabolite analyses. (**A**) Overview of how the discovery cohort and external ecological and metabolite data sets were used. Each data set was limited to the questions supported by its metadata, and unsupported claims are shown explicitly. (**B**) Summary of the strongest inference supported for the D15–D28 trajectory, antibiotic association, serum metabolite alignment, external ecology, and fecal metabolite findings. (**C**) External ecological results, showing median ECS and median putative SCFA-associated abundance in the discovery cohort and external ecological support sets. (**D**) Paired fecal metabolite changes from baseline to onset in PRJNA1124986. These fecal metabolite results are exploratory, and the external data sets provide ecological and metabolite context rather than independent clinical-event validation.

Across cohorts, median ECS values were −0.202 in discovery, 1.516 in PRJNA526551, and 0.361 in PRJNA352060, while the internally defined disruption-state rates were 9.4%, 8.9%, and 20.5%, respectively. Prespecified genera absent from an external table were represented by zero-filled columns only to preserve the fixed formula. Differences in cohort composition, primer region, laboratory processing, and taxonomic classification preclude direct prevalence comparisons. PRJNA526551, therefore, supports only application of the fixed formulas and description of score distributions, while incomplete PRJNA352060 phase labels preclude formal longitudinal replication.

PRJNA1124986 was retained as an exploratory fecal metabolite support set (Fig. S6). In 17 paired baseline/onset comparisons, the SCFA module changed in the hypothesized direction (median delta, −0.215), but the paired Wilcoxon test was not significant (*P* = 0.174). TCA-related and lactate/succinate modules also had negative median deltas with nonsignificant paired tests. These results provide directional context only and are not confirmatory evidence.

## DISCUSSION

This study characterizes an operational and potentially reversible gut ecological disruption state during AML therapy. Putative SCFA-associated genera, pathobiont abundance, domination, ECS, and community diversity showed their largest descriptive shifts during D15–D28. Joint modeling substantially changed the interpretation of that timing: after adjustment for antibiotic exposure during the preceding 0−14 days, the D15–D28 contrast attenuated toward the null, whereas recent exposure retained a consistent association with disruption. D15–D28 should, therefore, be viewed as a clinically recognizable window in which ecological disruption and antibiotic exposure accumulate together, not as an exposure-independent therapy-stage effect.

Compared with prior AML and hematologic-cancer microbiome studies emphasizing diversity loss, infectious complications, or pathogen domination, ECS integrates two opposing ecological features into a continuous sample-level summary (2, 4, 13). This summary can be aligned with exposure windows and metabolite measurements, so its value is organizational rather than mechanistic or predictive. Patient-level cross-fitting and bootstrap resampling showed that the D1–D7 to D15–D28 contrast was not created solely by estimating scaling parameters in the same patients. However, direct clinical comparison did not show stable incremental information from ECS beyond Shannon loss and maximum pathobiont abundance. Shannon loss also showed the stronger nominal association with subsequent C. difficile infection. These data, therefore, do not support presenting ECS as superior to established metrics.

The ecological state is best interpreted as a compositional shift away from genera associated in prior literature with anaerobic SCFA-producing communities and toward prespecified pathobionts. This interpretation is consistent with colonization-resistance biology and pathogen domination after microbiome disruption (6, 22–25), but the genus set does not measure SCFA production and the pathobiont set is not a diagnostic pathogen assay. Agreement between fixed ECS and a prespecified compositional log-ratio in the discovery and external genus tables shows that the summary is not dependent on a single abundance-aggregation scheme. Because both metrics use the same taxa, this agreement is a compositional robustness check rather than independent biological validation.

Serum butyrate/isobutyrate and selected indole-related metabolites remained associated with stool ecological indices after adjustment for treatment window, reducing concern that pooled correlations were explained entirely by their shared D15–D28 trajectory. Although microbial metabolites can influence epithelial and immune physiology (26, 27), these serum measurements remain systemic readouts influenced by host metabolism, diet, renal and hepatic function, treatment, and microbial processes. The results do not show that the profiled stool organisms produced the serum metabolites, nor do they establish pathway activity or metabolic flux. The paired fecal-metabolite result from PRJNA1124986 was directionally compatible but nonsignificant and remains exploratory.

The joint models directly address the near-collinearity between treatment day and accumulating antibiotic exposure. Recent antibiotic-days retained conditional associations with lower putative SCFA-associated abundance, higher pathobiont burden, and higher ECS, while the D15–D28 coefficients no longer clearly differed from D1–D7. Working-correlation and patient-bootstrap analyses supported this pattern, as did the inverse-probability-of-observation sensitivity analysis. The temporal ordering of exposure before each stool sample strengthens the exposure definition but does not identify causality: antibiotics are administered in response to prophylaxis protocols, fever, infection concern, mucosal injury, neutropenia severity, and other time-varying clinical factors (1, 2). Residual confounding by indication and disease severity is, therefore, likely. A recent AML study likewise linked antibiotic administration patterns to microbiome disruption and subsequent antibiotic-resistant infection or colonization events (28), reinforcing the need to model exposure timing while not resolving confounding by indication.

The internally derived trajectory strata summarize repeated ecological measurements at patient level, but their thresholds were derived from cohort-specific percentiles. Differences in their component metrics are consequently self-referential. The lagged 7- and 14-day analyses provide a less circular clinical anchor and did not show stable ECS associations or FDR-significant metric comparisons. Strata A, B, C, and no threshold signal are descriptive labels and must not be used to imply discrimination, calibration, severity, or risk classification without prospective evaluation using frozen thresholds.

The external analyses show that the fixed ecological formulas can be applied to other genus-level tables and yield concordant sample ordering between ECS and the compositional log-ratio. They do not constitute external validation. Primer region, sequencing workflow, taxonomic classification, cohort composition, and missing prespecified genera can shift score distributions. Neither external microbiome data set contained the linked treatment-day, antibiotic, metabolite, and clinical-event structure required to replicate the discovery analysis. The external results, therefore, support application of the fixed formulas and distributional concordance, not independent biological or clinical validation.

Several limitations remain. First, this observational analysis cannot separate antibiotic effects from time-varying indication, disease severity, chemotherapy, neutropenia, nutrition, or mucosal injury. Second, 16S amplicon profiles, even when denoised to ASVs, cannot resolve strains, gene content, virulence, antimicrobial resistance, or metabolic function; genus-level aggregation further limits resolution. Third, serum metabolomics do not substitute for paired fecal metabolomics, and nearest-day pairing is imperfect. Fourth, PostD28 inference rests on only 24 samples from 18 patients, so apparent recovery applies only to observed samples. Fifth, the observation-weight analysis cannot recover eligibility after discharge, death, transfer, or protocol completion. Sixth, event counts and follow-up metadata limit clinical modeling, and the study was not designed to validate prediction. Finally, relative-abundance measures remain compositional despite the fixed log-ratio sensitivity analysis (20, 29, 30).

The practical implication is that D15–D28 is a high-yield sampling window for prospective study of antibiotic-coupled ecological disruption, not an independent ecological phase. Future studies should freeze the genus sets, ECS formula, compositional handling, sampling schedule, antibiotic windows, follow-up eligibility, and clinical endpoints before enrollment. Shotgun metagenomics, absolute microbial load, paired fecal and serum metabolomics, and adequately powered external cohorts would be required for strain-level, functional, causal, or predictive claims.

## MATERIALS AND METHODS

### Cohorts and analytical roles

The analysis used PRJNA451154 as the discovery cohort because it contained longitudinal stool microbiome profiles that could be linked to treatment-day metadata, antibiotic exposure, serum metabolomics, and patient-level clinical-event metadata (5). The primary stool microbiome analysis was restricted to samples labeled as adequate in the stool-sample metadata table. This filter yielded 508 stool samples from 67 patients.

We specified the more limited uses of the external data sets before analysis. PRJNA352060 was used as an external explicit-stool ecological sensitivity set, and PRJNA526551 was used as an external genus-profile ecological support set and for genus-informed proxy analyses (4). PRJNA1124986 was used only to examine the direction of patient-linked fecal metabolite changes (31). These external data sets were not pooled with PRJNA451154 for clinical-event, antibiotic, or AML treatment-stage inference because they did not contain the same linked treatment-day, antibiotic, and clinical-event metadata.

### 16S processing and genus-level abundance tables

Adequate stool samples from PRJNA451154 were processed in QIIME 2 2026.4 using batched workflows (32). Manifests were built from the adequate-sample accessions, and each batch was imported as paired-end Phred33 FASTQ data. Because the QIIME 2 Deblur denoise-16S workflow supports only forward reads, the imported paired-end data were internally converted to single-end input and only forward reads were denoised at a trim length of 250 nt to generate amplicon sequence variants (ASVs) (33). Batch-level ASV feature tables and representative sequences were merged without sequence-similarity clustering.

Merged ASV representative sequences were assigned taxonomy against a SILVA 138 V4 515F/806R reference (34). The V4 reference reads were generated with primers GTGYCAGCMGCCGCGGTAA and GGACTACNVGGGTWTCTAAT, with a minimum length of 200 nt and maximum length of 500 nt. Taxonomy was assigned with QIIME 2 classify-consensus-vsearch using 97% sequence identity and 80% query coverage (35), followed by taxonomic aggregation at genus level. Thus, 97% identity was used only for taxonomic assignment, not to cluster reads into operational taxonomic units. This distinction was verified from the preserved executable pipeline and derived tables; QIIME 2 artifact-level provenance could not be re-extracted because the intermediate .qza files were unavailable during the revision audit.

Exported genus-level BIOM tables were converted into sample-by-genus relative abundance tables. Taxonomic labels were parsed by extracting the g field, removing brackets, and assigning empty, unassigned, or uncultured labels to Unclassified. Genus counts with the same cleaned label were summed. Sample-level relative abundances were computed by dividing each genus count by the total genus-level count in that sample.

### Fixed ecological indices

Ecological indices were computed from genus-level relative abundance tables using fixed genus sets. The putative SCFA-associated genus set contained *Faecalibacterium, Roseburia, Subdoligranulum, Anaerostipes, Coprococcus, Butyricicoccus, Blautia, Eubacterium, Agathobacter,* and *Lachnospira*. The pathobiont set contained *Enterococcus, Streptococcus, Escherichia-Shigella, Klebsiella, Enterobacter, Pseudomonas, Staphylococcus,* and *Proteus*.

For each sample, putative SCFA-associated abundance was the summed relative abundance of the prespecified putative SCFA-associated genera. Pathobiont abundance was the corresponding sum over the pathobiont set, and maximum pathobiont abundance was the highest relative abundance among those genera. Domination30 was defined as maximum pathobiont abundance of at least 0.30. These genus sets are ecological proxies and do not measure metabolite production, virulence, or strain identity.

The ecological composite score was defined as log10(pathobiont abundance +1e−6) minus log10(putative SCFA-associated abundance +1e−6). The primary binary ecological-disruption label combined domination and depletion of the putative SCFA-associated genus signal. Depletion was defined as abundance less than or equal to the internal discovery-cohort 25th percentile (0.00110620278833313). Ecological disruption was coded as present when Domination30 and the depletion criterion were both met. This operational state was treated as potentially reversible and descriptive rather than as irreversible ecosystem failure.

A compositional sensitivity analysis used the difference between mean natural-log abundances of the 8 pathobiont and 10 putative SCFA-associated genera, with a discovery-frozen zero replacement of 1.06316248312e−05 (30). The cross-fitted ECS was constructed from three axes: −log10(putative SCFA-associated abundance +1e−6), log10(pathobiont abundance +1e−6), and maximum pathobiont abundance. Patients were assigned to fivefold. Within each training set, each axis was summarized at the patient level and standardized using the median as the center and the median absolute deviation multiplied by 1.4826 as the scale. These scaling parameters were applied only to held-out patients; standardized axis values were truncated to [−5, 5] and averaged with equal weights. Thus, all 508 samples from 67 patients received a held-out score. The cross-fitted ECS is an internal sensitivity construction and differs from the fixed ECS; only the fixed ECS formula was frozen for external application. Period contrasts used each patient’s within-window median and 2,000 patient-level bootstrap resamples among the 52 patients observed in both D1–D7 and D15–D28.

### Treatment-period windows

Sample days were defined relative to day 1 of chemotherapy. Samples were assigned to five mutually exclusive treatment windows: PreChemo for days before day 1, D1–D7 for days 1–7, D8–D14 for days 8–14, D15–D28 for days 15–28, and PostD28 for days after day 28. The same period labels were used for ecological trajectory summaries, patient-clustered models, diversity analysis, genus-informed proxy analysis, and figure generation.

### Antibiotic exposure variables and models

Antibiotic exposure was derived from the antibiotic exposure table. Start and stop days were converted to numeric days relative to chemotherapy day 1. Active antibiotic courses on the stool sampling day were counted when the antibiotic interval covered the sample day. Cumulative antibiotic exposure up to a stool sample was computed with inclusive day counting across all antibiotic intervals ending on or before the sample day or overlapping it.

Antibiotic class variables were derived by name and route. Anti-pseudomonal beta-lactams included cefepime, piperacillin-tazobactam, meropenem, imipenem, ceftazidime, and aztreonam. Glycopeptides were identified by vancomycin. Metronidazole and fluoroquinolones were identified by drug name, with fluoroquinolones including levofloxacin, ciprofloxacin, and moxifloxacin. Oral/gut-active exposure included oral-route antibiotics and vancomycin, metronidazole, or fidaxomicin. Any intravenous exposure was assigned when the route was intravenous.

Lagged antibiotic exposure windows were anchored to each stool sample. The windows were 0–3, 0–7, 0–14, and 7–14 days before the sample day. For each antibiotic class and window, exposure days were computed by interval overlap using inclusive day counting. The primary recent-exposure variable was total antibiotic-days during the preceding 0–14 days and was scaled per 7 antibiotic-days for interpretation. We quantified the unadjusted coupling between treatment window and recent exposure using one-way eta-squared, defined as the proportion of total sample-level exposure variation attributable to between-window differences.

Repeated stool samples were analyzed with Gaussian generalized estimating equations (GEE) using patient as the independent cluster, exchangeable working correlation, and robust sandwich covariance (36). For log-transformed putative SCFA-associated abundance, log-pathobiont burden, fixed ECS, and cross-fitted ECS, we fitted treatment-window-only models, recent-antibiotic-only models, and joint models containing both terms; D1–D7 was the period reference. Independence working correlation and 2,000 patient-level bootstrap refits were sensitivity analyses. All coefficient tables report observations and independent patient clusters. To assess informative observation, we modeled whether an eligible sample was followed by another stool sample within 7 days and then fitted patient-period models with stabilized inverse-probability-of-observation weights estimated from period, prior observation and ecology, baseline age and sex, and preceding clinical events. We truncated weights at their observed 1st and 99th percentiles and compared weighted with unweighted joint models.

### Diversity and community-structure analysis

Alpha diversity was computed from sample-normalized genus relative abundance matrices. Shannon diversity was calculated as −sum(p_i * log[p_i + 1e−12]), Simpson diversity as 1 − sum(p_i^2^), richness as the number of genera with relative abundance at least 0.1%, and effective genera as exp(Shannon). Period-level alpha-diversity differences were tested with Kruskal-Wallis tests across treatment windows and two-sided Mann-Whitney tests comparing D15–D28 with D1–D7 and PreChemo.

Community structure was assessed with Bray-Curtis distances computed from genus relative abundance profiles. Principal coordinates analysis was performed by classical double-centering of the Bray-Curtis distance matrix. Period-level PERMANOVA used 999 permutations under unrestricted and within-patient restricted schemes, with fixed random seeds of 20260611 and 20260612, respectively (37). In the restricted analysis, treatment-period labels were permuted only among samples from the same patient. Beta-dispersion summaries were computed as distances from each sample to its period centroid in the first five PCoA dimensions.

### Serum-stool metabolome pairing and metabolite associations

Serum metabolome samples from PRJNA451154 were paired to the nearest stool ecology sample from the same patient by absolute day difference. The prespecified primary window was 7 days (243 pairs from 35 patients), with a 3-day sensitivity window (227 pairs from 35 patients). For each analysis, one nearest stool sample was assigned to each serum sample under the fixed pairing rule.

Candidate metabolites were identified before modeling from annotation fields using pathway terms for short-chain fatty acids, fatty acids, bile acids, tryptophan/indole, glutamate, polyamines, the TCA cycle, and branched-chain amino acids. Each metabolite and ecological metric was rank inverse-normal transformed in its full source cohort. Primary period-adjusted associations used Gaussian GEE of metabolite rank score on ecological rank score and treatment window, with patient as the independent cluster, independence working correlation, and robust sandwich covariance. The primary family comprised all 276 prespecified candidate metabolites crossed with fixed ECS and log-transformed putative SCFA-associated abundance; Benjamini-Hochberg correction was applied across all 552 models (38). Exchangeable working correlation, the 3-day pairing window, and within-period Spearman correlations were sensitivity analyses.

### Descriptive patient trajectories and lagged clinical-event anchoring

Patient-level ecological trajectories were derived descriptively from repeated sample-level indices. Patients with fewer than three stool samples or fewer than two sampled periods were assigned to the sparse-data category. Remaining eligible patients were assigned to Stratum A, Stratum B, Stratum C, or no threshold signal using internally derived percentiles of maximum ECS and minimum putative SCFA-associated abundance together with maximum pathobiont abundance and domination/disruption indicators. Because both thresholds and strata were defined in PRJNA451154, these labels were not evaluated as severity, diagnostic, or prognostic classes.

For lagged clinical anchoring, outcomes were the first recorded C. difficile infection, diarrhea, or neutropenic fever within 7 or 14 days after each stool sample. Samples collected on or after a patient’s first event day were excluded for that outcome. Cross-fitted ECS, fixed ECS, Shannon loss, and maximum pathobiont abundance were rank inverse-normalized and oriented, so higher values indicated disruption. Primary logistic GEE used independence working correlation, patient-clustered robust covariance, and treatment-window adjustment; 2,000 patient-level bootstrap resamples estimated intervals for metric effects. Benjamini-Hochberg correction covered the 24 primary event-horizon-metric comparisons. Exploratory incremental models added ECS to models already containing Shannon loss and maximum pathobiont abundance and assessed coefficient stability and change in quasi-likelihood under the independence model criterion. These analyses evaluated association and incremental ecological information, not predictive discrimination.

### External ecological and metabolite analyses

PRJNA352060 explicit stool samples (*n* = 365) and PRJNA526551 genus profiles (*n* = 447) were represented as genus-level relative-abundance tables and analyzed with the discovery-frozen 10 putative SCFA-associated genera, 8 pathobiont genera, ECS pseudocount, compositional zero replacement, Domination30 cutoff, and discovery thresholds (4). Prespecified genera absent from a table were represented by zero-filled columns solely to preserve the formula. No genus set, pseudocount, zero replacement, coefficient, or threshold was recalibrated externally. External analyses evaluated whether the fixed rules could be applied, described score distributions, and assessed ECS/log-ratio agreement. They did not test treatment-stage, clinical-event, strain-level, or functional validity.

PRJNA1124986 was analyzed as an exploratory patient-linked fecal metabolite support set (31). Structured supplement tables were used to identify patient-level baseline and onset metabolite profiles for the PA15-0780 candidate cohort. Metabolite values were log10-transformed after adding 0.1 and then *z*-scored. Baseline-to-onset paired deltas were computed for each patient with both time points. Paired Wilcoxon signed-rank tests were used when applicable.

### Exploratory genus-informed ecological guild proxy

The exploratory genus-informed proxy grouped observed genera into curated ecological guilds labeled putative SCFA-associated, tryptophan/indole-associated, bile-acid-associated, anaerobic mucus-barrier-associated, pathobiont inflammatory, and oxygen-tolerant expansion groups. Guild values were summed genus relative abundances. The contrast was log10(pathobiont inflammatory plus oxygen-tolerant expansion abundance + 1e−6) minus log10(putative SCFA-associated plus anaerobic mucus-barrier abundance + 1e−6). The labels describe literature-informed ecological associations only. This analysis is not PICRUSt2, shotgun metagenomics, metatranscriptomics, a direct functional assay, or evidence of pathway activity.

### Software and reproducibility

Microbiome processing was performed with QIIME 2 (version 2026.4), Deblur, VSEARCH, and the SILVA 138 reference database. Statistical analyses, ecological-index calculations, sensitivity analyses, and figure generation were performed in Python using pandas, NumPy, SciPy, statsmodels, scikit-bio, matplotlib, and seaborn. Generalized estimating equation models were fitted with statsmodels. Multiple-testing correction used the Benjamini-Hochberg false-discovery-rate procedure. Analysis code and derived machine-readable tables are publicly available in Zenodo (https://doi.org/10.5281/zenodo.21696652).

## Supplementary Material

Reviewer comments

## Data Availability

All raw sequencing data analyzed in this study are publicly available in the NCBI Sequence Read Archive under BioProject accessions PRJNA451154, PRJNA352060, PRJNA526551, and PRJNA1124986. All derived data sets and analysis scripts generated in this study, including genus-level abundance tables, ecological index tables, model output tables, metabolite association matrices, patient-level trajectory assignments, external-support summary tables, and scripts for data processing, statistical modeling, sensitivity analyses, and figure generation, are publicly available in Zenodo (https://doi.org/10.5281/zenodo.21696652).

## SUPPLEMENTAL MATERIAL

Supplemental figures spectrum.02578-26-s0001.docx Fig. S1 to S6.

Supplemental legends spectrum.02578-26-s0002.docx Legends for Fig. S1 to S6.

Table S1 spectrum.02578-26-s0003.xlsx Sample and patient contributions across treatment windows in the PRJNA451154 discovery cohort.

OPEN PEER REVIEW reviewer-comments.pdf An accounting of the reviewer comments and feedback.

ASM does not own the copyrights to Supplemental Material that may be linked to, or accessed through, an article. The authors have granted ASM a non-exclusive, world-wide license to publish the Supplemental Material files. Please contact the corresponding author directly for reuse.
