# Supplementary material for: Longitudinal ecological disruption and metabolite associations of the gut microbiome during acute myeloid leukemia therapy
Source: Microbiol Spectr. 2026 Sep 8;14(10):e02578-26. doi: 10.1128/spectrum.02578-26 (PMC13689431; doi:10.1128/spectrum.02578-26)
Supplement: Supplementary file 1 [file reviewer-comments.pdf]

ASM | Microbiology Spectrum

Longitudinal ecological collapse and metabolic coupling of the gut microbiome during acute myeloid leukemia therapy

Jian Zhou, Rong Li, Mengyue Zhang, Yong Wu, and Rong Hu

Corresponding Author(s): Rong Hu, Fujian Medical University

Review Timeline:

Submission Date:	July 31, 2026
Editorial Decision:	August 6, 2026
Revision Received:	August 7, 2026
Accepted:	August 8, 2026

Editor: Jan Claesen

Reviewer(s): The reviewers have opted to remain anonymous.

Transaction Report:

(Note: With the exception of the correction of typographical or spelling errors that could be a source of ambiguity, letters and reports are not edited. The original formatting of letters and referee reports may not be reflected in this compilation.)

DOI: https://doi.org/10.1128/spectrum.02578-26

Re: Spectrum02578-26 (**Longitudinal ecological collapse and metabolic coupling of the gut microbiome during acute myeloid leukemia therapy**)

Dear Dr. Rong Hu:

Thank you for the privilege of reviewing your work, and thanks for carefully addressing the Reviewers' comments from your prior submission to mSystems. I am pleased to inform you that your manuscript has been editorially accepted for publication. However, there are a few additional questions in the submission form that need to be answered before your manuscript is officially accepted. Once these are completed, please return your submission so that I can move your paper forward to acceptance.

Please return the manuscript within 60 days; if you cannot complete the submission questions within this time period, please contact me.

Thank you for submitting your paper to Spectrum.

Sincerely,
Jan Claesen
Editor
Microbiology Spectrum

Responses to Reviewers

Longitudinal ecological disruption and metabolite associations of the gut microbiome during acute myeloid leukemia therapy

Dear Editor and Reviewers,

We thank the editor and reviewers for their careful evaluation of the manuscript. We have revised the manuscript substantially and provide a point-by-point response below. The principal revisions include joint modeling of treatment window and recent antibiotic exposure; patient-clustered robust inference and bootstrap sensitivity analyses; treatment-window-adjusted metabolite analyses; direct comparison of ECS with Shannon diversity and maximum pathobiont abundance; explicit sample and patient counts; patient-level cross-fitting, informative-observation sensitivity analysis, and fixed compositional analyses; clarification of ASV generation; and systematic softening of causal, predictive, and validation language.

Editorial requirements

E.1

Comment: Please provide a compare PDF file that indicates the changes from the original submission (by highlighting or underlining the changes). Please upload this PDF file separately from the manuscript file as file type "Marked Up Manuscript - For Review Only File."

Response: A separate comparison PDF has been prepared from the original submitted manuscript. New or revised passages are shown with yellow highlighting and underlining. This file is provided separately as "Marked Up Manuscript - For Review Only File."

E.2

Comment: To expedite the review process, please provide a Response to Reviewers file that includes the reviewers' comments and gives a point-by-point response to each of the issues raised by the reviewers. Please upload this file separately from the manuscript file with the object title "Responses to Reviewers" and the file class type "Response to Reviewer Comments."

Response: This document reproduces the supplied reviewer comments and responds to each issue point by point. It is provided separately with the object title "Responses to Reviewers" for upload as "Response to Reviewer Comments."

E.3

Comment: This may or may not be applicable to your manuscript. If your study entailed generation of any high-throughput sequencing or other large datasets that are routinely deposited in online repositories (eg. GEO database), please make sure that your paper has a "Data Availability" Section. Please set your datasets to public and list the accession numbers in this section. This will speed up the editorial process at Microbiology Spectrum.

Response: The revised manuscript contains a dedicated Data availability section. It lists the NCBI Sequence Read Archive BioProject accessions PRJNA451154, PRJNA352060, PRJNA526551, and PRJNA1124986 for the raw data. It also lists the public Zenodo record for the derived datasets and analysis code (https://doi.org/10.5281/zenodo.21696652). The same Zenodo DOI is now stated consistently in the Software and reproducibility section.

Response to Reviewer 1

R1.1

Comment: Time versus antibiotic exposure are not disentangled. The D15-D28 peak coincides with the expected neutropenic nadir and with maximal cumulative antibiotic exposure. Because cumulative exposure is near-collinear with treatment day, the current models cannot separate a "therapy-period ecological state" from a simple dose-response to accumulating antibiotics. Please add models that include treatment window and lagged/recent antibiotic exposure jointly, and report whether the D15-D28 signal persists after adjustment for recent exposure (and vice versa).

Response: We agree that treatment time and antibiotic exposure needed to be modeled jointly. We therefore added patient-clustered GEE models containing both treatment window and antibiotic exposure during the preceding 0-14 days. In the joint fixed-ECS model, the D15-D28 versus D1-D7 coefficient attenuated to 0.251 (95% CI, -0.372 to 0.874), whereas recent antibiotic exposure remained associated with higher ECS (0.722 per 7 antibiotic-days; 95% CI, 0.529 to 0.915). The corresponding D15-D28 contrasts for the putative SCFA-associated signal and pathobiont burden were also attenuated, while recent exposure retained conditional associations with both outcomes. We now interpret D15-D28 as a descriptive window in which ecological disruption and antibiotic exposure accumulate together, rather than as an exposure-independent therapy-period state. These changes appear in Results ("The D15-D28 ecological contrast attenuated after adjustment for recent antibiotic exposure"), Materials and Methods ("Antibiotic exposure variables and models"), Figure 3, and the Discussion.

R1.2

Comment: Implausibly extreme p-values in the class-level GEE models. Coefficients such as P=8.29e-21 and P=1.77e-14 are difficult to reconcile with ~508 repeated samples from only 67 patients. These raise concerns about residual pseudoreplication, working-correlation misspecification, or an inappropriate variance estimator. Please confirm that robust standard errors were used, report the number of independent clusters driving each estimate, and consider reporting effect sizes with more emphasis than these p-values, which are likely uninterpretable at face value.

Response: We agree that the original presentation overemphasized nominal P values and did not make the independent sampling unit sufficiently clear. All revised GEE models use patient as the independent cluster and robust sandwich covariance. The principal models include 508 observations from 67 independent patient clusters, and the revised model tables report both observation and cluster counts. We now emphasize coefficients and robust 95% confidence intervals rather than extreme nominal P values. We also added sensitivity analyses using an independence working correlation and 2,000 patient-level bootstrap refits. The revised specification and reporting are described in Materials and Methods ("Antibiotic exposure variables and models") and Results ("The D15-D28 ecological contrast attenuated after adjustment for recent antibiotic exposure"), with the principal effect estimates displayed in Figure 3.

R1.3

Comment: ECS is defined from SCFA-related taxa and pathobiont abundance, and serum SCFA metabolites are then correlated with SCFA-related taxa and with ECS. Some of these associations are

close to definitional. Moreover, the correlations are not adjusted for treatment window, so shared temporal structure (both metabolites and ecology shifting during D15-D28) could inflate them. Please provide within-period or period-adjusted (e.g., partial Spearman / mixed-model) metabolite-ecology associations to show the coupling is not simply a time confounder.

Response: We agree that pooled correlations could reflect shared treatment-period structure and that ECS associations partly inherit the taxa used in the score. We replaced the unadjusted emphasis with treatment-window-adjusted, patient-clustered GEE analyses of rank-normalized variables and retained within-period Spearman correlations as a sensitivity analysis. In the primary 7-day serum-stool pairing, butyrate/isobutyrate remained associated with the putative SCFA-associated genus signal (coefficient, 0.476; 95% CI, 0.309 to 0.643) and fixed ECS (-0.372; 95% CI, -0.516 to -0.227) after treatment-window adjustment. Similar estimates were obtained with the 3-day pairing window. The text now explicitly states that these are ecological-metabolite associations and cannot establish microbial production, metabolic flux, or causality. The changes appear in Results ("Serum metabolite associations persisted after treatment-window adjustment"), Materials and Methods ("Serum-stool metabolome pairing and metabolite associations"), Figure 4, Figure S5, and the Discussion.

R1.4

Comment: The individual components, diversity loss, anaerobe/SCFA depletion, Enterococcus/Enterobacteriaceae domination, antibiotic-driven disruption, are well established. The claimed advance is the integration into a time-structured composite phenotype. Please sharpen the Discussion to state concretely what the ECS/collapse framework predicts or reveals that the constituent metrics do not, ideally with a direct comparison (e.g., does ECS add information beyond Shannon diversity or max-pathobiont alone for the clinical anchoring in Fig. 6?).

Response: We agree that the contribution of ECS should be evaluated directly against established component metrics. We therefore compared cross-fitted ECS, fixed ECS, Shannon loss, and maximum pathobiont abundance for first recorded C. difficile infection, diarrhea, or neutropenic fever during the subsequent 7 or 14 days, with treatment-window adjustment and patient-level bootstrap intervals. None of the 24 primary comparisons met an FDR threshold of 0.05. In exploratory incremental models, adding ECS to Shannon loss and maximum pathobiont abundance did not provide stable incremental information. We have consequently revised the Discussion to describe ECS as an integrative organizational summary, not a superior or validated predictor. These analyses appear in Results ("Clinical anchoring analyses showed no incremental information from ECS"), Materials and Methods ("Descriptive patient trajectories and lagged clinical-event anchoring"), Figure 6, and the Discussion.

R1.5

Comment: The Severe/Moderate/Mild thresholds are derived from internal percentiles and then used to show group differences in the same cohort, including the CDiff/diarrhea associations. This is internally consistent but self-referential. Please state explicitly that these are descriptive strata (you partly do), and temper any language implying discriminative value.

Response: We agree. We replaced the severity labels with neutral labels (Strata A, B, and C), state

explicitly that the thresholds and strata were derived in the same cohort, and describe the resulting differences as self-referential descriptive summaries. We do not interpret these strata as severity, discrimination, calibration, diagnosis, prognosis, or risk classification. Clinical events are now examined separately in lagged analyses that do not use the internally defined strata as predictors. These revisions appear in Results ("Clinical anchoring analyses showed no incremental information from ECS"), Materials and Methods ("Descriptive patient trajectories and lagged clinical-event anchoring"), Figure S3, and the Discussion.

R1.6

Comment: Per-window sample sizes are not reported. Given the strong reliance on window comparisons (Figs 2, 5, 6), please add n per window and per patient, and note how many patients contribute to PostD28, since the recovery interpretation may rest on few samples.

Response: We added both sample and patient counts for every treatment window: PreChemo, 29 samples from 25 patients; D1-D7, 140 from 62; D8-D14, 122 from 61; D15-D28, 193 from 56; and PostD28, 24 from 18. Patients contributed a median of 8 stool samples (range, 1-14), and the patient-level distribution is reported in Table S1. We also revised the PostD28 interpretation to state that the apparent partial recovery is based on a small, selected subset and does not establish cohort-wide recovery. These changes appear in Results ("The discovery and external datasets were assigned distinct analytical roles" and "Ecological disruption metrics showed a descriptive peak during D15-D28") and in the Discussion limitations.

R1.7

Comment: "Ecological collapse" terminology is strong. Given that PostD28 shows partial recovery and the state is reversible, consider whether "disruption" or "vulnerable ecological state" is more accurate, or define "collapse" operationally early on.

Response: We agree and have replaced "ecological collapse" with "ecological disruption" in the title and throughout the manuscript. The state is now defined operationally as a potentially reversible compositional condition, and recovery language is restricted to the observed PostD28 samples. The Abstract, Importance, Introduction, Results, Materials and Methods ("Fixed ecological indices"), figures and legends, and Discussion have all been revised accordingly.

Response to Reviewer 2

R2.1

Comment: I would like to thank the authors for the opportunity to review this manuscript. I appreciate the considerable time and effort invested in conducting this research and preparing the manuscript. There are two major issues with the manuscript, that I think are fundamental, and that prevents me from approving. The first is that the findings are all based on 16S rRNA gene sequencing. The 16S rRNA gene is, intrinsically, limiting in resolving power. Additionally, while the authors utilise QIIME2, they cluster sequences using search at 97% sequencing similarity (i.e., defining OTUs), the field has moved onto using ASVs instead of OTUs.

Response: We agree that 16S rRNA gene sequencing has intrinsic limits and have strengthened this limitation throughout the revised manuscript. The analysis is restricted to genus-level ecological associations and does not support strain-level identification, gene content, virulence, antimicrobial-resistance, or functional claims. We also audited and clarified the sequence-processing workflow. The reads were denoised with QIIME 2 Deblur denoise-16S at a trim length of 250 nt to generate amplicon sequence variants (ASVs); no 97% sequence-similarity clustering was used to define OTUs. The 97% identity threshold was used only by classify-consensus-vsearch for taxonomic assignment against the SILVA reference before genus-level aggregation. We revised Materials and Methods ("16S processing and genus-level abundance tables") to make this distinction explicit and added the corresponding limitations to the Abstract, Importance, figure legends, and Discussion.

We appreciate the reviewers' comments, which led to a more rigorous and appropriately bounded manuscript.

Re: Spectrum02578-26R1 (**Longitudinal ecological collapse and metabolic coupling of the gut microbiome during acute myeloid leukemia therapy**)

Dear Dr. Rong Hu:

Thanks for addressing the Spectrum-specific items in the submission site. Your paper has now been officially accepted for publication. Congratulations!

Your manuscript has been accepted, and I am forwarding it to the ASM production staff for publication. Your paper will first be checked to make sure all elements meet the technical requirements. ASM staff will contact you if anything needs to be revised before copyediting and production can begin. Otherwise, you will be notified when your proofs are ready to be viewed.

Data Availability: ASM policy requires that data be available to the public upon online posting of the article, so please verify all links to sequence records, if present, and make sure that each number retrieves the full record of the data. If a new accession number is not linked or a link is broken, provide production staff with the correct URL for the record. If the accession numbers for new data are not publicly accessible before the expected online posting of the article, publication may be delayed; please contact ASM production staff immediately with the expected release date.

Publication Fees: For information on publication fees and which article types have charges, please visit our website. We have partnered with Copyright Clearance Center (CCC) to collect author charges. If fees apply to your paper, you will receive a message from no-reply@copyright.com with further instructions. For questions related to paying charges through RightsLink, please contact CCC at ASM_Support@copyright.com or toll free at +1-877-622-5543. CCC makes every attempt to respond to all emails within 24 hours.

ASM Membership: Corresponding authors may join or renew ASM membership to obtain discounts on publication fees. Need to upgrade your membership level? Please contact Customer Service at Service@asmusa.org.

Embargo Policy: A press release may be issued as soon as the manuscript is posted on the Spectrum Latest Articles webpage. The corresponding author will receive an email with the subject line "ASM Journals Author Services Notification" when the article is available online.

Cover Image Submissions: If you would like to submit a potential Cover Image, please email a file and a short legend to spectrum@asmusa.org. Please note that we can only consider images that (i) the authors created or own and (ii) have not been previously published. By submitting, you agree that the image can be used under the same terms as the published article. Image File requirements: TIF/EPS, 7.5 inches wide by 8.25 inches tall (at least 2,250 pixels wide by 2,475 pixels tall), minimum 300 dpi resolution (600 dpi preferred), RGB, and no figure elements, e.g., arrows or panel labels. The legend should be a short description of the image, 1-2 sentences recommended. Please use the ASM Journals Cover Image Sizing Tool (https://journals.asm.org/pb-assets/html/asm-cover-sizing-tool.html) to ensure that your proposed cover image meets our size requirements.

The ASM Journals program strives for constant improvement in our submission and publication process. Please tell us how we can improve your experience by taking this quick Author Survey.

Thank you for submitting your paper to Spectrum.

Sincerely,
Jan Claesen
Editor
Microbiology Spectrum
