# Supplementary material for: Longitudinal ecological disruption and metabolite associations of the gut microbiome during acute myeloid leukemia therapy
Source: Microbiol Spectr. 2026 Sep 8;14(10):e02578-26. doi: 10.1128/spectrum.02578-26 (PMC13689431; doi:10.1128/spectrum.02578-26)
Supplement: Supplementary file 2 [file spectrum.02578-26-s0001.docx]

**Supplementary Figures and Legends**

**Figure S1.** Patient-level cross-fitting and bootstrap stability of ECS. (A) Sample-level agreement between fixed ECS and fivefold patient-level cross-fitted ECS, with points colored by treatment window. Robust centers and scales were estimated in training patients and applied only to held-out patients; all 508 samples from 67 patients received held-out scores. (B) Within-patient D15–D28 minus D1–D7 contrasts for fixed and cross-fitted ECS among the 52 patients observed in both windows. Points are observed contrasts and lines are 95% confidence intervals from 2,000 patient-level bootstrap resamples. (C) Bootstrap distributions of the paired contrasts for the two scores. Cross-fitting addresses reuse of patients in scaling-parameter estimation but does not remove time-varying confounding.

**Figure S2.** Sensitivity to informative stool observation. (A) Estimated probabilities of observing a subsequent stool sample within 7 days among 179 observed eligible patient-periods. (B) Distributions of raw stabilized inverse-probability-of-observation weights and weights trimmed at the observed 1st and 99th percentiles. (C) Unweighted and trimmed-weight estimates of the D15–D28 versus D1–D7 contrast for the putative SCFA signal, pathobiont burden, and fixed ECS. (D) Corresponding coefficients per 7 recent antibiotic-days. Points show coefficients and lines show robust 95% confidence intervals. This proxy analysis cannot recover eligibility after discharge, death, transfer, or protocol completion.

**Figure S3.** Internally derived descriptive patient strata. (A) Median fixed ECS trajectories by treatment window for Strata A, B, C, and no threshold signal. Lines show stratum medians and shaded regions show interquartile ranges. (B) Corresponding trajectories of maximum pathobiont abundance after log10 transformation with a 0.0001 offset. (C) Number of patients in each stratum according to the window in which the internally defined threshold signal peaked. Thresholds and strata were derived in the same cohort; they are descriptive summaries, not validated severity, discrimination, calibration, or prediction categories.

**Figure S4.** Compositional log-ratio sensitivity and application of fixed formulas to external datasets. (A) Distribution of the prespecified mean-log pathobiont-minus-SCFA contrast across discovery treatment windows. (B) Sample-level concordance between fixed ECS and the fixed compositional log-ratio in PRJNA451154, PRJNA526551, and explicit stool samples from PRJNA352060. (C) Proportions meeting discovery-frozen low-SCFA, high-ECS, and operational disruption-state thresholds in the three datasets. The same genus sets, pseudocount, zero replacement, and thresholds were used without external recalibration. Agreement between metrics that use the same taxa is a compositional robustness check, not independent biological or clinical validation.

**Figure S5.** Serum-stool pairing-window sensitivity. (A) Numbers of nearest serum-stool pairs and contributing patients by treatment window for the primary within-7-day pairing. (B) Treatment-window-adjusted rank-score coefficients for butyrate/isobutyrate, indoleacetate, 3-indoxyl sulfate, and anthranilate with the putative SCFA signal and fixed ECS using pairing within 3 versus 7 days. Points show coefficients and lines show patient-clustered robust 95% confidence intervals.

**Figure S6.** Exploratory patient-linked fecal metabolite support in PRJNA1124986. Baseline and onset z-scored module values are shown for SCFA, lactate/succinate, TCA-related, and 2-hydroxyglutarate modules among 17 patients with both time points. Boxes summarize the distributions and points show individual values; displayed P values are from paired Wilcoxon signed-rank tests. These data provide directional context only and are not confirmatory evidence of ecological-metabolic coupling in the discovery cohort.
